# Relation of the degree of obesity in childhood to adipose tissue insulin resistance

**DOI:** 10.1007/s00592-018-01285-3

**Published:** 2019-01-12

**Authors:** Emilia Hagman, Omri Besor, Karen Hershkop, Nicola Santoro, Bridget Pierpont, Mariana Mata, Sonia Caprio, Ram Weiss

**Affiliations:** 1 Department of Human Metabolism and Nutrition, Braun School of Public Health, Hebrew University, Jerusalem, Israel; 2 Department of Clinical Science, Intervention and Technology, Karolinska Institutet, Blickagången 6A, 141 57 Stockholm, Sweden; 3 Department of Pediatrics, Ruth Rappaport Children’s Hospital, Rambam Medical Center, Haifa, Israel; 4 Department of Pediatrics, Yale University, New Haven, CT USA

**Keywords:** Pediatric obesity, Insulin resistance, Fatty acid, Adipose tissue

## Abstract

**Aims:**

In this study, we investigated whether adipose tissue insulin resistance (IR) is affected by the degree of obesity during the fasting and post-prandial state, independent of glucose tolerance among obese children and adolescents. We also tested whether systemic subclinical inflammation is associated with adipose tissue IR.

**Methods:**

Subjects were recruited to the Yale Pathophysiology of Type 2 Diabetes in Youth Study (NCT01967849). An oral glucose-tolerance test was performed to establish glucose-tolerance status and blood samples were drawn for measurement of free fatty acids (FFAs), to calculate the area under the curve (AUC) of FFA. Adipose tissue insulin resistance was calculated as the product of insulin and FFA concentrations.

**Results:**

In total, 671 children and adolescents (58.6% females) were included with a mean age of 13.3(2.7) years and BMI *Z* score of 2.45(0.31). The degree of obesity emerged as an independent predictor of both fasting and post-prandial adipose IR, *p* < 0.0001. Higher degree of obesity was associated with greater AUC FFA (lower suppression) compared to lower degree of obesity, *p* = 0.01. Furthermore, higher levels of IL-6 were positively associated with post-prandial adipose tissue IR, *p* = 0.02.

**Conclusions:**

The degree of obesity in childhood and adolescence is strongly associated with adipose tissue IR independent of glucose tolerance. This is reflected not only in calculated indices of adipose IR but also in lower suppression of FFAs during the OGTT regardless of glucose tolerance or fasting adipose tissue IR. Furthermore, markers of subclinical inflammation such as IL-6 are associated with adipose tissue IR, independent of other factors.

**Electronic supplementary material:**

The online version of this article (10.1007/s00592-018-01285-3) contains supplementary material, which is available to authorized users.

## Introduction

Insulin resistance is a reduction in the response to insulin of specific tissues and results in reduced inhibition of lipolysis in adipose tissue, which, in turn, contributes to a reduced skeletal muscle glucose uptake and an increased hepatic glucose production [1]. We have previously shown that, in children with obesity, the presence of an adverse metabolic phenotype is independently affected by both the degree of obesity and by a reduction of whole-body insulin sensitivity [2]. Adipose tissue is an endocrine organ that affects both glucose and lipid metabolism by releasing adipokines, pro-inflammatory factors, and free fatty acids (FFAs), all of which affect glucose metabolism and alter insulin signaling [3]. Altered glucose tolerance in children and adolescents with obesity is associated with greater resistance to insulin in adipose tissue [4], yet the relation of adipose insulin sensitivity, independent of whole-body insulin sensitivity, and the degree of obesity in the pediatric age group are less clear.

Indices describing the sensitivity of adipose tissue to insulin have been developed using fasting as well as post-prandial insulin and free fatty acid concentrations [5]. Adipose tissue release of FFA via unsuppressed lipolysis is the main manifestation of tissue insulin resistance (IR). Circulating FFAs play a major role in the development of reduced insulin sensitivity [6, 7] and beta-cell dysfunction [8], both via lipotoxicity. It is, thus, crucial to evaluate the sensitivity of adipose tissue to insulin specifically in the prandial state to study the delicate interplay of circulating FFAs with insulin secretion and action. While fasting indices of adipose insulin resistance have been studied in obese children [4, 9, 10], little is known about the prandial state, specifically in this context of obesity-related insulin resistance. Infiltration of adipose tissue by macrophages is probably one of the drivers of adipose IR playing a critical role in the establishment of the chronic inflammatory state and metabolic dysfunction [11], which are commonly detected in children with obesity and may be exacerbate during the developmental stage of puberty [12, 13].

It has recently been shown that adipose tissue IR in children and adolescents [4] as well as adults [5], defined as reduced suppression of FFAs during the oral glucose-tolerance test (OGTT), is associated with prediabetes and diabetes and with an altered adipocytokine profile. Moreover, it has been shown that obese compared to non-obese children and adolescents has reduced insulin sensitivity of adipose tissue [9], particularly in the fasting state. In the current analysis, we expanded this fundamental observation and performed additional analyses to investigate if, among obese children and adolescents, adipose tissue IR is affected by the degree of obesity during the fasting and post-prandial state, independent of glucose tolerance. We also investigate whether systemic subclinical inflammation, reflected by C-reactive protein (CRP) and interleukin-6 (IL-6) concentrations, is associated with adipose tissue IR. In other words, we attempted to link obesity per-se and subclinical inflammation with fasting and post-prandial adipose IR while removing the effect of glucose tolerance, a factor that has previously been shown to confound these relations.

## Methods

### Subjects

Subjects were recruited to the Yale Pathophysiology of Type 2 Diabetes in Youth Study, a long-term, multiethnic cohort aimed at studying the early alternations in glucose metabolism in obese children and adolescents (NCT01967849). Subjects were eligible to participate if they were at the age of 7–20 years and had a body mass index (BMI) that exceeded the 95th centile for age and sex [14]. Following subject assent and parental consent, a complete medical history and physical examination were performed. Weight was measured using a scale (Tanita Corp), and height was measured in triplicate with a wall-mounted stadiometer. Obesity categories according to age and sex-specific BMI percentiles were defined as: class I obesity (≥ 95th percentile to < 120% of the 95th percentile), class II obesity (≥ 120–< 140% of the 95th percentile), class III obesity (≥ 140–< 160% of the 95th percentile), and class IV obesity (≥ 160% of the 95th percentile) [12].

An oral glucose-tolerance test (OGTT, 1.75 g/kg body weight up to 75 g) was performed to establish glucose-tolerance status. Subjects were studied at the Yale Pediatric Clinical Center at 08:00 am after a 10-h overnight fast as previously reported [15]. Ranges of 2-h glucose were categorized into four groups: 1: ≤100 mg/dl, 2: 101–120 mg/dl, 3: 121–140 mg/dl, and 4 > 140 mg/dl at 2 h following glucose ingestion.

Blood samples were drawn for the measurement of free fatty acids (FFAs) at 0, 30, 60, and 120 min. All blood samples were immediately put on ice, centrifuged for 30 min, and stored at − 80 °C. FFAs samples were allowed to clot first, and then, serum was separated per the instructions of the assay manufacturer. All subjects tested negative for autoimmune markers for type 1 diabetes (insulin antibody, GAD65, and islet cell antibody 512). The study was ethically approved by the Human Investigations Committee of the Yale School of Medicine.

### Analytical methods

Plasma glucose was determined using YSI 2700 Analyzer (Yellow Springs Instruments). Plasma insulin and total adiponectin levels were measured using double antibody RIAs (Millipore inc. insulin intra- and interassay coefficients of variation are 6.8 and 11.6%, respectively; adiponectin intra- and interassay coefficients of variation are 7.1 and 9.5%, respectively). C-reactive protein (CRP) was measured using the ultrasensitive assay (Kamiya Biomedical). For CRP, the intra-assay coefficient of variation is no greater than 3.0%, and the interassay coefficient of variation is no greater than 11.6%. IL-6 was measured using a highly sensitive solid-phase ELISA (R&D Systems) (lower limit of detection: 0.16 pg/ml; intra- and interassay coefficients of variation: 7.4 and 7.8%, respectively). Plasma FFAs were determined using an enzymatic colorimetric method assay for the quantitative determination of non-esterified fatty acids in serum (Wakochem, Ind.). Plasma leptin levels were measured using an RIA assay from Linco (leptin intra- and interassay coefficients of variation: 6.5 and 8.0%, respectively).

### Calculations

Insulin sensitivity was calculated using the OGTT-derived whole-body insulin sensitivity index (WBISI, also known as the Matsuda index) [16]. WBISI is based on the values of insulin (microunits/ml) and glucose (mg/dl) obtained throughout the OGTT and the corresponding fasting values [16]. The fasting adipose insulin resistance (Adipo-IR) was calculated as the product of fasting insulin and FFA concentrations [17]. An additional index of adipose insulin resistance in the prandial state was calculated using the mean of the product of fasting insulin and FFA concentrations at time 0, 30, 60, and 120 min at the OGTT [5]. Area under the curve (AUC) of FFAs during the OGTT, as means to express FFA suppression during the study, was calculated using the trapezoidal rule.

### Statistical analysis

Data are presented as means ± standard deviations. The primary outcome of the analysis was the prandial adipose tissue insulin resistance index. The variables Adipo-IR, WBISI, CRP, and IL-6 values were log-transformed using the natural logarithm before analysis. Crude analyses were performed using the analysis of variance or the Chi-square test where appropriate. In adjusted analyses for the primary and secondary aims, linear regression models were applied. Independent variables in these models included sex, age, race, 2 h glucose category, and degree of obesity category. Sensitivity analyses were performed including only NGT subjects. The analysis was performed using the SAS Statistical software (version 9.4, SAS Institute Inc, Cary, NC, USA).

## Results

### Study participants (Table 1)

**Table 1:** Study participants by obesity categories

	Obesity class I, *n* = 123 (18.3%)	Obesity class II, *n* = 246 (36.7%)	Obesity class III, *n* = 174 (25.9%)	Obesity class VI, *n* = 128 (19.1%)	*χ*^2^/ANOVA
Females (%)	64.4	60.2	58.6	50.0	0.12
Caucasian/African-American/Hispanic	65.0/17.1/17.9	50.0/33.3/16.7	43.1/34.5/22.4	33.6/43.0/23.4	< 0.0001
Age (years)	13.5 ± 2.7	13.5 ± 2.7	13.2 ± 2.7	13.1 ± 2.9	0.40
BMI *Z* score	1.98 ± 0.15	2.36 ± 0.13	2.61 ± 0.11	2.85 ± 0.17	< 0.0001
Fasting glucose (mg/dl)	91.0 ± 7.4	92.3 ± 9.5	92.8 ± 7.6	94.3 ± 9.6	0.02
2 h glucose (mg/dl)	120.6 ± 23.5	121.7 ± 28.4	119.7 ± 25.1	126.9 ± 30.7	0.12
2 h glucose category^a^	17.1/38.2/30.9/13.8	17.9/37.0/26.0/19.1	21.3/35.1/27.0/16.7	12.5/35.2/31.2/21.1	0.64
Fasting Adipo-IR (mmol/l × µU/ml)	14.7 ± 8.7	17.4 ± 11.8	20.6 ± 10.9	25.8 ± 17.0	< 0.0001
Prandial Adipo-IR (mmol/l × µU/ml)	31.6 ± 27.1	35.5 ± 30.1	39.5 ± 24.3	49.5 ± 41.4	< 0.0001
WBISI	2.16 ± 1.35	1.80 ± 0.98	1.56 ± 0.85	1.53 ± 0.88	< 0.0001
CRP (mg/l)	1.85 ± 3.79	2.75 ± 3.91	4.01 ± 5.87	6.44 ± 6.44	< 0.0001
IL-6 (pg/ml)	2.07 ± 2.95	2.26 ± 1.69	2.56 ± 1.93	4.24 ± 3.18	< 0.0001
Leptin (ng/ml)	19.9 ± 9.8	28.6 ± 11.6	34.6 ± 13.8	43.5 ± 15.4	< 0.0001
Adiponectin (µg/ml)	7.8 ± 4.0	7.9 ± 4.1	7.3 ± 3.4	7.5 ± 3.9	0.45

Obesity class I represents 100–120%, class II: 120–140%, class III: 140–160%, and class IV ≥ 160% of the 95th percentile

*BMI* body mass index, *WBISI* whole-body insulin sensitivity index

^a^2 h glucose categories correspond to 1: ≤ 100 mg/dl, 2: 101–120 mg/dl, 3: 121–140 mg/dl, 4 > 140 mg/dl at 2 h after glucose ingestion on the OGTT

In total, 671 children and adolescents participated in the analysis with a mean age of 13.3 (2.7) years. The majority of participants were females (58.6%) and the race distribution was 47.8% Caucasian, 32.5% African-American, and 19.7% Hispanic. The BMI *Z* score was 2.45 (0.31) and ranged between 1.65 and 3.50. The distribution of participants within the degree of obesity categories was 18.3%, 36.7%, 25.9%, and 19.1% in obesity categories 1, 2, 3, and 4, respectively. Of note, the rate of Caucasians decreased, while the rate of African-Americans and Hispanics increased with greater degrees of obesity.

Both indices of adipose tissue IR (fasting and prandial) increased with the degree of obesity (*p* < 0.0001), while whole-body insulin sensitivity (WBISI) decreased with degree of obesity (*p* < 0.0001). No differences in 2 h glucose between obesity categories were detected (*p* = 0.64).

### Predictors of adipose tissue insulin resistance (Table 2)

**Table 2:** Linear regression models for predictors for adipose tissue insulin resistance (AT-IR) and WBISI

	ln fasting AT-IR		ln prandial AT-IR		ln WBISI	
	Estimate	*p*	Estimate	*p*	Estimate	*p*
Sex (male vs. female)	− 0.06	0.16	0.07	0.16	0.05	0.23
Age	− 0.01	0.29	0.01	0.20	− 0.01	0.44
Race (AA vs. Caucasians)	0.02	0.75	0.11	0.07	− 0.04	0.43
Race (Hispanic vs. Caucasians)	0.03	0.57	0.02	0.81	− 0.10	0.08
2 h glucose category	0.19	< 0.0001	0.21	< 0.0001	− 0.28	< 0.0001
Obesity class II vs. class I	0.17	0.003	0.15	0.042	− 0.14	0.018
Obesity class III vs. class I	0.37	< 0.0001	0.32	< 0.0001	− 0.28	< 0.0001
Obesity class IV vs. class I	0.53	< 0.0001	0.42	< 0.0001	− 0.28	< 0.0001
Estimates for adipose tissue insulin resistance and WBISI in NGT subjects (*n* = 551)						
Sex (male vs. female)	− 0.05	0.24	0.06	0.27	0.06	0.17
Age	− 0.003	0.73	0.02	0.025	− 0.01	0.06
Race (AA vs. Caucasians)	0.02	0.66	0.13	0.042	− 0.04	0.41
Race (Hispanic vs. Caucasians)	0.08	0.18	0.06	0.43	− 0.15	0.0130
2 h glucose category	0.16	< 0.0001	0.20	< 0.0001	− 0.24	< 0.0001
Obesity class II vs. class I	0.13	0.036	0.13	0.10	− 0.07	0.24
Obesity class III vs. class I	0.34	< 0.0001	0.32	0.0002	− 0.25	0.0001
Obesity class IV vs. class I	0.50	< 0.0001	0.45	< 0.0001	− 0.25	0.0007

Categories for 2 h glucose represent ≤ 100 mg/dl, 101–120 mg/dl, 121–140 mg/dl, and > 140 mg/dl

Obesity class I represents 100–120%, class II: 120–140%, class III: 140–160%, and class IV ≥ 160% of the 95th percentile

*WBISI* whole-body insulin sensitivity index, *AA* African-American

**Fig. 1:**
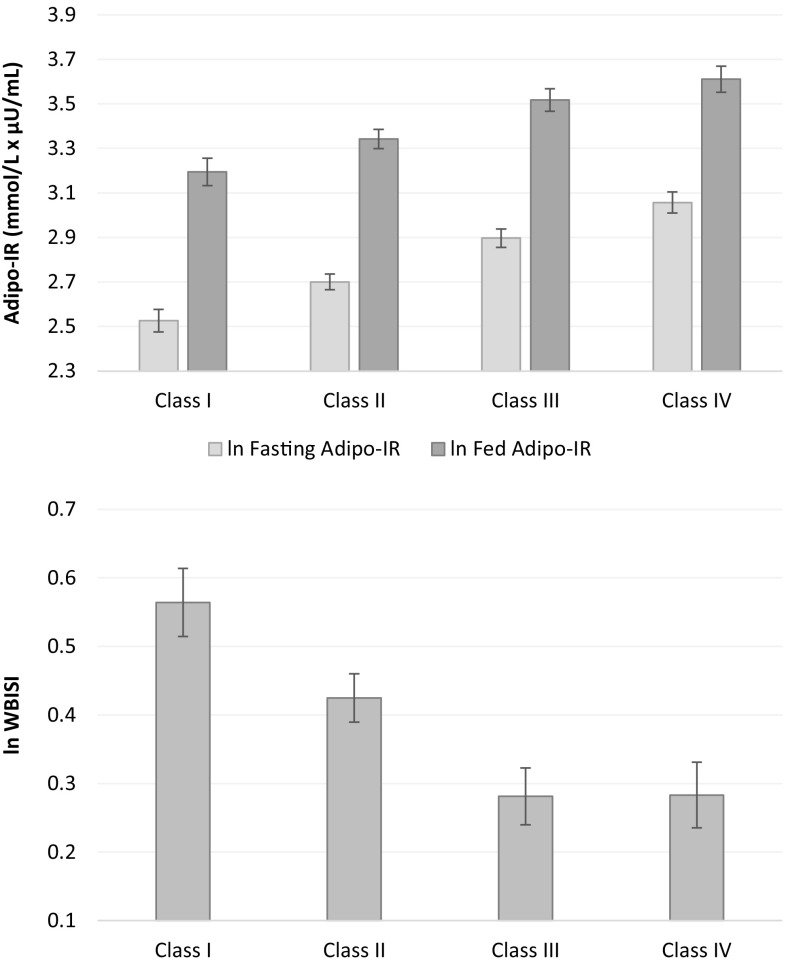
Adipose insulin resistance and whole-body insulin sensitivity across obesity categories adjusted for sex, age, race, and 2 h glucose category. Data are presented as adjusted means and SE. *p* for trend are < 0.0001 in all the analyses

We identified the predictors of adipose tissue IR using linear regression models where the adipose IR index was used as the dependent variable, and sex, age, race, 2 h glucose category, and the degree of obesity were used as independent variables. In these models, the degree of obesity (as reflected by obesity categories) emerged as an independent significant predictor of both indices of adipose insulin resistance (ln fasting and prandial Adipo-IR). Specifically, rising degrees of obesity were associated with greater fasting and prandial adipose IR as well as with lower whole-body insulin sensitivity (Fig. 1). Of note, in agreement with the previous findings, glucose tolerance, assessed using the 2 h glucose category even within the normal range, was also an independent significant predictor of Adipo-IR.

Limiting the analysis to subjects with NGT (to examine these findings regardless of alterations in glucose metabolism known to affect the outcomes) only mildly attenuated these results and the degree of obesity remained a significant predictor of adipose insulin resistance in the models.

Limiting the analysis to subjects with data on leptin (*n* = 658) and adiponectin (*n* = 662) revealed in adjusted models that higher levels of adiponectin were significantly associated with lower fasting Adipo-IR (*β* = − 0.03, *p* < 0.0001) and post-prandial Adipo-IR (*β* = − 0.04, *p* < 0.0001), while the degree of obesity remained a significant predictor of adipose insulin resistance in both models. Leptin was not independently associated with Adipo-IR indices (data not shown).

### Relation of the post-prandial FFA profile and the degree of obesity

**Fig. 2:**
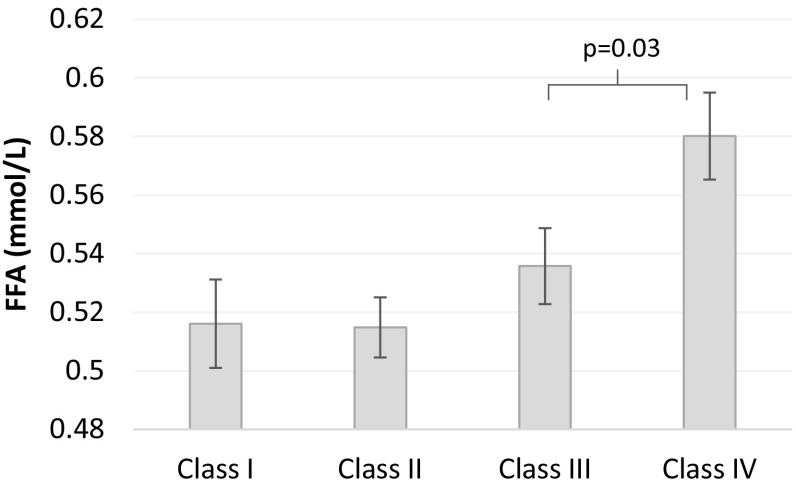
Levels of fasting free fatty acids by obesity category, *p* for trend = 0.0007

Fasting FFAs increased across obesity categories (*p* for trend = 0.0007). However, only in subjects with class IV obesity, fasting FFAs were significantly greater than the other obesity categories (*p* = 0.03, Fig. 2). After adjustment for baseline FFA, no differences in post-prandial FFA were observed between obesity categories (*p* = 0.22). Of note, boys had 0.015 mmol/l higher fasting FFA levels than girls. (*p* = 0.02). There were no statistical significant differences in other fasting or 120′ glucose or FFA levels between the sexes within each obesity category (data not shown). Levels of glucose, insulin, and free fatty acids (FFA) during oral glucose-tolerance test divided by obesity class I–IV are provided in Supplementary Fig. 1.

**Fig. 3:**
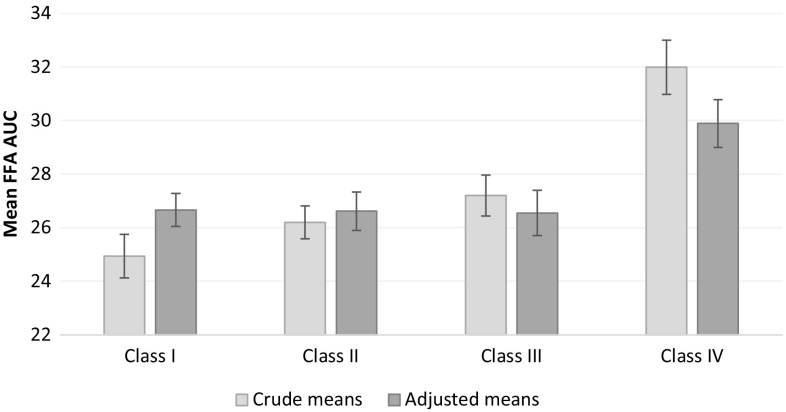
Crude and adjusted means (SE) for the area under the curve of free fatty acids (FFA) during 2-h oral glucose-tolerance test by obesity categories. Higher values represent lower suppression of FFAs. *p* for trend for crude and adjusted means are < 0.001 and 0.0149 respectively

The suppression of FFAs during the OGTT was expressed as AUC of FFAs, where greater values represent lower suppression. We compared the AUC of FFA during the OGTT between obesity categories while adjusting for sex, age, race, 2 h glucose category, and fasting adipose tissue IR. Obesity class IV had a higher AUC FFA (lower suppression) compared to class I (*p* = 0.01, Fig. 3). The FFA AUC was also higher in boys, increased with fasting Adipo-IR, and with age within the full sample. Upon limiting the analysis to those with NGT, obesity class IV still had higher AUC FFA than obesity class I.

### Relation of adipose tissue insulin resistance and markers of inflammation

To test the relation of inflammatory markers and adipose tissue insulin resistance, we first tested simple correlations of adipose insulin resistance indices and IL-6/CRP. Later, we incorporated IL-6 and CRP separately into the models described earlier where indices of adipose IR were the dependent variables.

In crude analyses, both (ln)CRP and (ln)IL-6 were positively associated with (ln)fasting Adipo-IR (*p* = 0.003 and 0.0001, respectively) and with (ln) fed Adipo-IR (*p* = 0.01 and 0.0004 respectively). (ln)WBISI was not associated with (ln)CRP (*p* = 0.08) or (ln)IL-6 (*p* = 0.06).

Investigating the effect of CRP and IL-6 on adipose IR indices after adjusting for the same covariates as in the models presented in Table 2, revealed that higher levels of IL-6 were positively associated with (ln) post-prandial Adipo-IR, *β* = 0.11, *p* = 0.02. No significant effect of CRP was found in adjusted models (data not shown).

## Discussion

Obesity is associated with whole-body insulin resistance yet this resistance may differ between insulin responsive tissues and not always be directly related to the degree of obesity. Moreover, altered glucose tolerance is typically associated with insulin resistance in adolescents with obesity and may be a confounder when studying the relation of the degree of obesity, subclinical inflammation, and adipose IR. Our findings indicate that the degree of obesity in childhood and adolescence is strongly associated, independent of glucose tolerance, with adipose tissue insulin resistance. This is reflected not only in calculated indices of adipose IR but also in lower suppression of FFAs during the OGTT in subjects with the highest degree of obesity, regardless of glucose tolerance or fasting adipose tissue IR. Furthermore, we show that markers of subclinical inflammation such as IL-6 are associated with adipose IR, independent of other factors and regardless of glucose-tolerance status. These observations demonstrate for the first time in adolescents with obesity that the degree of obesity is a crucial component in the development of adipose IR, possibly mediated by inflammatory cytokines derived from fat depots.

Adipose tissue is a key player in modulation of energy balance in general and glucose metabolism in particular. Glucose metabolism is regulated by the interplay of insulin secretion and insulin sensitivity. While insulin sensitivity is typically referred to insulin responsive organs such as the liver in the fasting state and skeletal muscle in the post-prandial state, adipose tissue tends to be less appreciated in this context. The regulation of FFA release from adipose tissue governs FFA plasma concentrations, which in turn affects both insulin sensitivity and secretion directly (reducing both after prolonged exposure) [18, 19]. It has been shown that both FFAs concentrations and visceral fat are negatively associated with insulin secretion in obese adolescents [10]. Moreover, even overnight exposure to elevated FFAs administered exogenously has been shown to reduce both insulin sensitivity and indices of insulin secretion in obese adolescents [20, 21]. Metabolites such as acetyl CoA, that are derived from inflamed white adipose tissue lipolysis, are the main regulators of gluconeogenesis and thus of hepatic glucose production [22]. Consistent with our studies, others have also shown the marked differences of adipose IR between obese adolescents with normal vs. impaired glucose tolerance [4, 23]. In adults, the rise in adipose IR parallels that of beta-cell dysfunction in subjects with normal glucose tolerance prior to the development of overt diabetes [5]. These previous observations along with those presented herein suggest a role of lipotoxicity as a potential driver of altered glucose metabolism in pubertal adolescents.

Insulin resistance of the adipose tissue manifests mainly as reduced suppression of lipolysis and—to a lesser extent—in reduced glucose uptake [24]. Thus, understanding of adipose tissue IR independent of whole-body insulin sensitivity is of relevance to the understanding of glucose metabolism in obese youth. Our findings suggest that the resistance of adipose tissue to the effects of insulin is, indeed, directly associated with the degree of obesity, independent of glucose tolerance. This observation suggests that, above a certain amount of lipid storage, adipose tissue loses its capacity to continue the process of lipogenesis induced by ambient insulin concentrations at the same efficiency and rate that was present at lower amounts of adipose tissue mass. This is probably due to changes in cell size resulting in larger adipocytes, known to be less responsive to insulin and to be associated with altered glucose metabolism [25]. The reduced suppression of FFAs during the OGTT in those with greater degrees of obesity indicates that, above a certain adiposity threshold, both lower responsiveness to insulin resulting in non-suppressed lipolysis and greater FFA release as well as reduced FFA clearance (not evaluated in this analysis) result in greater circulating FFA concentrations that create lipotoxic effects and alter glucose metabolism [26, 27].

In this analysis, concentrations of IL-6 were positively related with the indices of adipose tissue insulin resistance. Moreover, even after adjusting for age, sex, ethnicity, glucose tolerance, and the degree of obesity, IL-6 remained a significant predictor of prandial adipose IR. The present analysis adds another piece to the puzzle of the effects of adiposity in childhood, and shows that the concentration of markers of inflammation in obese children is linked to their degree of obesity and glucose tolerance as well as to their degree of adipose tissue insulin resistance. The development of dysregulated fat characterized by infiltration of immune cells such as macrophages [28] and inducing a pro-inflammatory hormonal profile may be an early driver of both insulin resistance and beta-cell dysfunction in obese adolescents [29]. This suggests that adipose derived cytokines such as IL-6 may have direct effects on insulin responsive tissues such as the liver and muscle, affecting insulin signal transduction pathways in them as well as on the effects of insulin in the adipose tissue itself [30]. Thus, the interplay of total body fat on one hand and fat distribution on the other contributes to the overall metabolic phenotype of children with obesity via the effects of intracellular lipid transport/deposition and via pro-inflammatory cytokines respectively. Moreover, these observations highlight the potential mechanistic role of subclinical inflammation, independent of the degree of obesity and glucose tolerance, with insulin responsiveness of adipose tissue, and may shed light on the pathophysiology of dysmetabolism in the context of the other chronic inflammatory diseases in this age group.

In the present analysis, we used a large sample size and investigated a broad spectrum of degrees of obesity which allowed us to shed light on the relations of the degree of obesity and the metabolic function of adipose tissue in obese children. Our observations need further validation using tracing of FFAs and evaluation of their turnover in the face of changing insulin concentrations as well as in children with similar degrees of obesity yet different lipid partitioning patterns. Whether the sensitivity of subcutaneous compared to intra-abdominal fat to insulin in these children is different needs further investigation as well. There are some observations in the literature that a significant weight loss may improve adipose IR [9], yet the flexibility of the metabolic characteristics of adipose tissue in the face of weight changes (both gain and loss) and whether adipose IR may be modified without weight changes (such as by the use of thiazoladinediones [31]) in this population need further investigation.

## Electronic supplementary material

Below is the link to the electronic supplementary material.

Supplementary material 1 (DOCX 28 KB)
