# Supplementary material for: Relation of the degree of obesity in childhood to adipose tissue insulin resistance
Source: Acta Diabetol. 2019 Jan 12;56(2):219–26. doi: 10.1007/s00592-018-01285-3 (PMC6373259; doi:10.1007/s00592-018-01285-3)
Supplement: Supplementary file 1 — Supplementary material 1 (DOCX 28 KB) [file 592_2018_1285_MOESM1_ESM.docx]

Class IV

Class III

Class II

Class I

Supplementary Figure 1. Levels of glucose, insulin and free fatty acids (FFA) during oral glucose tolerance test, divided by Obesity Class I- IV.
